# Monte-Carlo simulation of the Siemens Artiste linear accelerator flat 6 MV and flattening-filter-free 7 MV beam line

**DOI:** 10.1371/journal.pone.0210069

**Published:** 2019-01-08

**Authors:** Alemeh Sadrollahi, Frank Nuesken, Norbert Licht, Christian Rübe, Yvonne Dzierma

**Affiliations:** Department of Radiotherapy and Radiation Oncology, Saarland University Medical Center, Homburg, Saarland, Germany; North Shore Long Island Jewish Health System, UNITED STATES

## Abstract

The aim of our work is to provide the up-to-now missing information on the Siemens Artiste FFF 7 MV beam line using a Monte-Carlo model fit to the realistic dosimetric measurements at the linear accelerator in clinical use at our department. The main Siemens Artiste 6MV and FFF 7MV beams were simulated using the Geant4 toolkit. The simulations were compared with the measurements with an ionization chamber in a water phantom to verify the validation of simulation and tuning the primary electron parameters. Hereafter, other parameters such as surface dose, spectrum, electron contamination, symmetry, flatness/unflatness, slope, and characteristic off-axis changes were discussed for both Flat and FFF mode. The mean electron energy for the FFF beam was 8.8 MeV and 7.5 MeV for Flat 6 MV, the spread energy and spot size of the selected Gaussian distribution source were 0.4 MeV and 1mm, respectively. The dose rate of the FFF beam was 2.8 (2.96) times higher than for the flattened beam for a field size of 10×10 (20×20) cm^2^. The electron contamination has significant contribution to the surface dose especially for the flattened beam. The penumbra, surface dose and the mean energy of photons decrease by removing the flattening filter. Finally, the results show that off-axis changes have no strong effect on the mean energy of FFF beams, while this effect was more considerable for the flattened beam.

## Introduction

Over the past years, flattening-filter-free (FFF) beams have gained increasing importance in radiotherapy treatment because of various advantages such as the higher dose rate and hence reduced treatment times, less head scatter and out-of-field dose, neutron production, and secondary cancer risk. Some properties have been controversely discussed, such as surface dose and spectral effects, which is mainly indebted to the fact that the most often used technical implementation (Varian linacs) applies identical accelerating energy, just leaving out the flattening filter, which results in a softening of the beam. In contrast to this, the Siemens implementation increases the incident electron beam energy for the FFF beam line to create closely similar depth-dose curves for the flat 6 MV and FFF 7 MV beams (“pdd-matched” beams).

Although Siemens left the market, there is still a large number of linear accelerators in use, many of which offer the FFF beam line. Only very little information is available on the dosimetric properties of this beam energy, mostly from the point of view of dosimetric measurements [1–3] and planning examples [4–12]. Detailed information about the beam softening-effects, surface dose, the electron contamination, spectral properties and off-axis changes can only be obtained by Monte Carlo modelling. To our knowledge, no detailed Monte-Carlo study of the Siemens FFF beam dosimetric properties has been presented in the literature, although a few studies have focused on the flat beam properties [13, 14, 15, 16, 17, 18, and 19]. Only one study has attempted to model the Siemens linac FFF beam [20]; however, they used literature data [21] to theoretically modify an existing model of a flat-beam Siemens KD accelerator (which does not offer FFF beams in reality) and presented a general analysis of beam quality specifiers rather than a model for the realistic FFF beam.

The aim of our work is therefore to provide the up-to-now missing information on the Siemens Artiste FFF 7 MV beam line using a Monte-Carlo model fit to the realistic dosimetric measurements at the linear accelerator in clinical use at our department [1]. The paramount dosimetric properties such as depth-dose curve, beam profiles, spectrum, electron contamination and surface dose are compared with the flat 6 MV beam line available at the same machine. In addition to this, further measures of quality (symmetry, flatness/unflatness, slope) are presented. Furthermore, characteristic off-axis changes are discussed, in particular the off-axis change in spectrum for the flat beam vs. the constant spectral properties in FFF mode. We hope that this study fills the gap of information on a beam line still widely in clinical use. Besides, the characteristic off-axis changes will be comparable to those observed at other linacs using an energy-matching approach for FFF beams and might therefore shed some light on the hitherto controversial issue.

## Material and method

### Simulation

**Fig 1:**
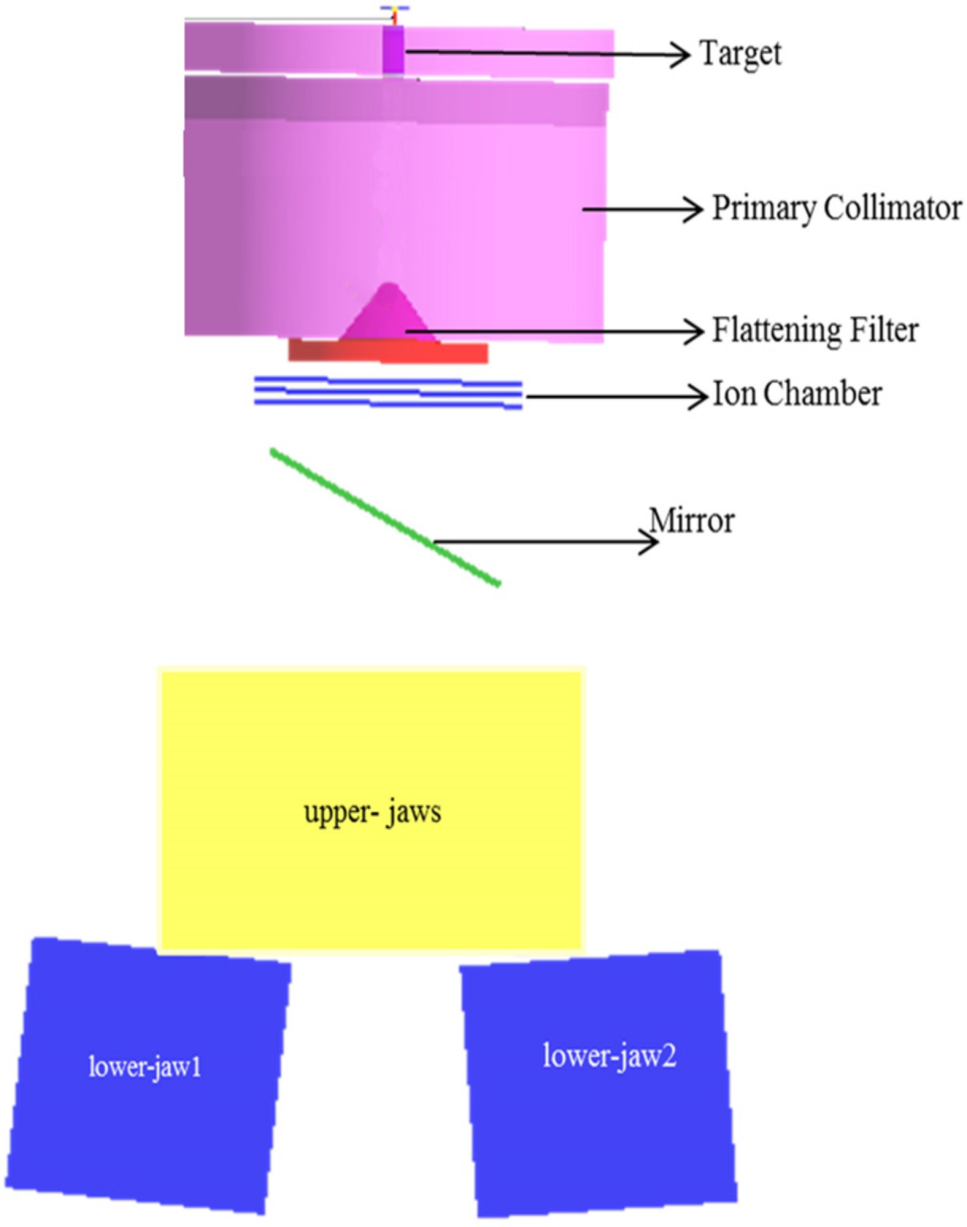
Cross-section view of the simulated treatment head of the Siemens Artiste in Flat 6MV mode by the Geant4 toolkit.

The flattening-filter-free beam energy 7 MV (FFF) of the Siemens Artiste medical linear accelerator and the corresponding 6 MV flat beam line were modelled using the Monte Carlo method. All simulations were performed by Geant4_10_03 [22] based on a system Intel Core i3-4130 CPU, 3.40 GHZ processor and a system Intel Core i3-2100 CPU, 3.10 GHZ processor. The geometry of the linac head relies on information from manufacturer references including target, primary collimator, and jaws; for the 6 MV beam, the flattening-filter was added to the aforementioned geometry. Overall, Fig 1 shows a complete simulation of the treatment head of the Siemens Artiste in Flat 6MV mode (visualization by OpenGL [23]).

All calculations were acquired for a water phantom of volume 40×40×40 cm^3^ at a source to surface distance (SSD) of 100 cm. The variance reduction technique lies at the heart of Monte Carlo simulations to increase the precision and improve the efficiency of a Monte Carlo code by reducing the calculation time [24]. Several variance reduction techniques have been proposed to improve the efficiency of MC simulation; cut-off energy and production threshold energy for secondary particles were applied as variance reduction techniques in this manuscript. Both parameters define a threshold energy for further transporting a particle. Whenever the energy of a particle falls below the energy cut-off, the particle is terminated and its energy is deposited locally. If the energy of the primary particle is below the production threshold, no secondary particle will be produced and the primary particle will be terminated by depositing the remaining energy locally, so that the particle history ends at this step. Energy cut-off and production threshold energy are both defined in terms of a travelling distance, which is internally converted into a corresponding energy for each material in Geant4. In our simulations, the range cut-off is defined as the default value (1 mm) for all materials and for gamma particles, electrons and positrons. Clearly, by increasing these thresholds, the computational time will be decreased.

Based on possible interactions in our simulation, the electromagnetic standard model option3 (emstandard_opt3) was used. This model can be applied to transport photons and charged particles in the range of energies between 10 eV and 100 TeV [25]. In this model, electromagnetic processes such as ionization, excitation, multiple scattering and bremsstrahlung radiation for charged particles, as well as Compton scattering, photoelectric effects and gamma transformations for photons are formulated. To avoid infrared divergence in electromagnetic interactions, some processes are required to remain above the production energy threshold of 1 mm as explained above.

The command-based-scoring method was used for the calculation of the dose distribution in the water phantom. In this method, the water phantom was voxelized to divide the volume into cells (voxels) of 5×5×5 mm^3^ to acquire the depth-dose curves and dose profiles and 1×1×1 mm^3^ for considering the buildup region and surface dose.

One of the important issues to be considered in Monte Carlo simulation is the statistical noise. In parcticular, with too small voxel dimension the statistical noise will increase considerably because of the randomness of scattered electron deposited energy [26]. In order to overcome these difficulties in Geant4, simulations can be improved by various methods: firstly, by using the variance reduction techniques outlined above; secondly, by calculating a higher number of histories for smaller voxel dimensions (which are defined by the required spatial resolution) [27]. However, increasing the number of histories will increase the computational time considerably. Therefore, a similar aim was achieved by using phase space files (PSF), which were placed before the jaws. After saving the particle information in this “intermediate step” in a PSF, this file can then subsequently be used as particle source in the next step of the simulation for scoring the photon beam characteristics in a water phantom. This method is known as virtual source model. The main advantage of this method is that the processing is faster than the classic Monte Carlo simulations and the statistical uncertainty in the dose calculation is increased by recycling incident particles (reading same particle many times) from the PSF [28,29,30]. Walters et al. [31] provide a general review of recycling and restarting phase-space particles.

### Measurements

The depth dose and profile beam data were measured in a PTW MP3 (PTW, Freiburg, Germany) water phantom using a Semiflex ionization chamber (PTW 31010) which has a sensitive volume of 2.75 mm^3^. All measurements for the field size of 10×10, 20×20 and 40×40 cm^2^ were performed at source-to-surface distance of 100 cm. Measurements were recorded in 1 mm steps using the PTW Verisoft system. The beam profiles were obtained for four different depths (1.9 cm and 10 cm for FFF 7XU; 1.6 cm and 10 cm for the flat 6X beam line). A detailed description of the measured dosimetric characteristics of the Siemens Artiste FFF and flat beam lines can be found in Dzierma et al. (2012) [1].

### Primary electron beam parameters

The validity of the Monte Carlo method depends on fine-tuning the electron beam parameters to the best match between measurements and simulation. In the present work, the mean energy, spread energy and spot size (FWHM) of the electron source were tuned by the Verhaegen and Seuntjens [32] method. In the FFF 7XU mode, the mean energy was selected in the range from 7 to 9 in 0.1 MeV steps and from 6 to 8 MeV in Flat 6MV mode for each independent run. The energy spread ranged from 100keV to 1MeV in 100 keV steps. The simulated beam has a 2D Gaussian distribution in the X-Y plane, with full width at half maximum (FWHM) ranging from 0.5 mm to 2mm.

### Photon beam characteristics

In order to calculate the depth-dose curves for FFF and Flat6MV beams, the total deposited dose (all particles) was calculated in voxels with dimension of 5×5×5 mm^3^ on the central axis via the command-base-scoring method. 1.5 × 10^9^ histories were taken into account and the PSF was recycled 5 times to have 7.5 ×10^9^ equivalent histories which were sufficient to attain acceptable statistical uncertainties (< 2%). The computations were performed on two computers (Intel Core i3-4130 CPU, 3.4 GHz processor and Intel Core i3-2100 CPU, 3.10 GHz processor) working separately, taking about 4 days for the creation of the PSF and … for each subsequent each simulation. Depth-dose curves were normalized to 100% at the depth of maximum dose. For analyzing the depth-dose curves, The D_20_/D_10_ (dose ratio at a depth of 20 cm to a dose at a depth of 10 cm) was determined to show the penetration and the dose rate of both configurations is defined as the dose per incident electron (Gy/primary electron) on the central axis at depth of 10 cm with SSD = 100 cm.

Beam profiles of the FFF beam and Flat6MV beam were calculated at a depth of 10 cm in the water phantom. Validation of the beam profiles was furthermore performed at the depth of maximum dose (D_max_), which was 19 mm for the FFF and 16 mm for the flattened beam. Similar to PDD curves, 1.5×10^9^ histories were calculated to achieve an average standard deviation below 2%. All flattened profiles were normalized to their respective maximum value on the central axis and the FFF beams were normalized according to Fogliata et al. [33] procedure (renormalization method, “shoulder point”). After normalization, the following parameters of the FFF profile were evaluated [33–35]:

- penumbra as the distance between the positions of the 20% and the 80% of the normalized profile,
- dosimetric field size as the distance between right and left inflection point,
- unflatness = D_CAX_/D_off-axis_ (D_CAX_ being the normalized dose at the central-axis and the D_off-axis_ the dose at the 80% field size)
- $Slope=\frac{D_{1}-D_{2}}{x_{1}-x_{2}}$ (x_1_ and x_2_ being two points at 2/3 and 1/3 of the half-profile, respectively, and D_1_ and D_2_ the normalized doses at x_1_ and x_2_, respectively),
- $peak\;position=\frac{I_{L}-I_{R}}{S_{L}-S_{R}}$ (I_L_ and I_R_ being the positions of left and right intercepts, S_L_ and S_R_ the left and right slope, respectively),
- symmetry as the maximum variation = (*D_x_*−*D_-x_*)_*max*_, in which D_x_ an D_-x_ are the normalized doses at x and–x positions.

Surface dose was defined as deposited energy in the first millimeter (the air-skin boundary) of the water phantom and the buildup region is defined from the surface of the phantom to depth of maximum dose in water phantom on central axis. It is noteworthy to mention that the calculated surface dose was obtained from a different program, in which voxels were defined with dimension of 1×1×1 mm^3^ to obtain the exact depth-dose in buildup region for 10 and 20 cm field side of FFF and Flat6MV beams.

### Photon spectra

Phase-space files recorded all information of secondary particles produced in the linac treatment head. These files at 100 cm from the target (on the surface of the water phantom) were used as sources to obtain the photon and electron fluence per incident electron for both FFF and flattened beams.

Additionally, to find the photon energy spectrum at a given position, the fluence of photons with different energies was calculated. To do this, the fluence was divided into intervals (bins) of 0.1 MeV and the result of the FFF and flattened beam were compared for two field sizes.

### Electron contamination

The contribution of electron contamination in the buildup region especially on surface dose cannot be neglected and should be fully evaluated using Monte Carlo computations. Geant4 has ability to estimate the electron contamination by killing the electron contamination above the water phantom, so the difference in surface dose from the full simulation and electron-kill gives the contribution of electron contamination to the total dose.

In this case, the surface dose was obtained in the first millimeter of the water phantom with 1×1×1 mm^3^ voxel size and the electron contaminant energy fluence at the surface of the water phantom also calculated in intervals of 0.1 MeV, for 10 and 20 cm field side of FFF and Flat6MV beams.

### Off-axis changes

The dependency of dosimetric properties such as depth-dose, penetration, surface dose and photon spectra on off-axis distance (OAD) for the FFF and Flat6MV beam was investigated. For calculating the dosimetric properties at various OADs, scoring planes were placed at three different OADs of 2.5 cm, 5cm, and 7.5 cm in three separate programs. In this work, *n* cm OAD will be taken to mean *n* cm shift along both the X and the Y directions, i.e. $\sqrt{2}\times n\;cm$ geometrical distance from the CAX. Similar to previous simulations, voxels with a dimension of 5×5×5 mm^3^ were defined for calculating the depth-dose; for the surface dose, the voxel size was 1×1×1 mm^3^. The photon fluence is obtained from a program in which a scoring plane with square shape was simulated around the CAX and off-axis with dimension 5×5 mm^2^. The range of energies from zero to 10 MeV was divided into 50 intervals of 0.5 MeV and then the number of photons passing the scoring plane were recorded and reported. The number of histories was set to 1×10^9^ and the open field size was 20×20 cm^2^.

## Results

### Primary electron beam parameters

**Fig 2:**
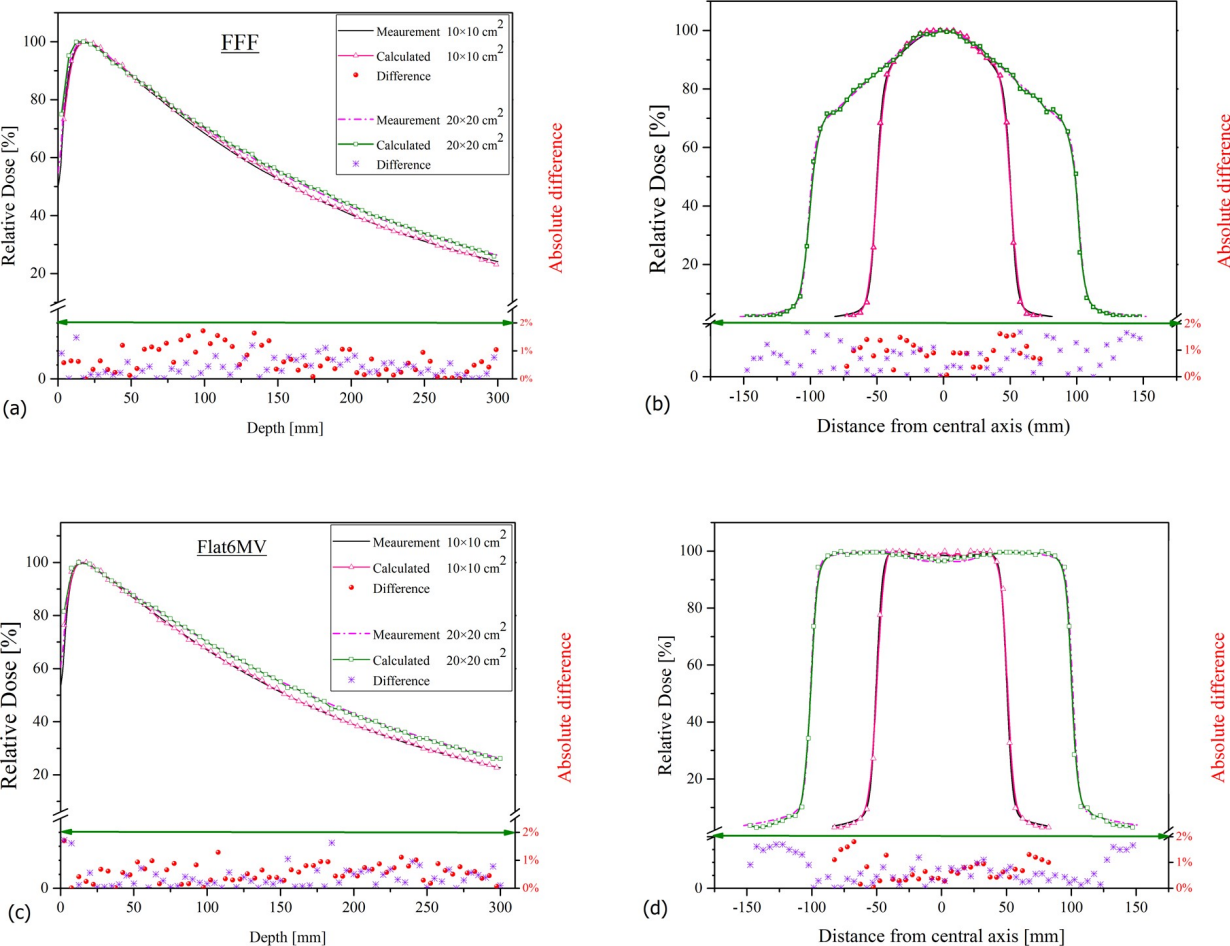
Validation of Monte Carlo simulation. a) depth-dose curve and b) beam profile of the FFF beams, c) depth dose curve and d) beam profile of the Flat6MV beam, for 10×10 and 20×20 cm^2^ field sizes in voxels with dimension of 5×5×5 mm^3^ at depth of maximum dose. Points and stars under the green line show the absolute difference between measurement and calculation. The Monte Carlo statistical uncertainty for each point is between 1% to 1.6% which is not shown in figures.

**Fig 3:**
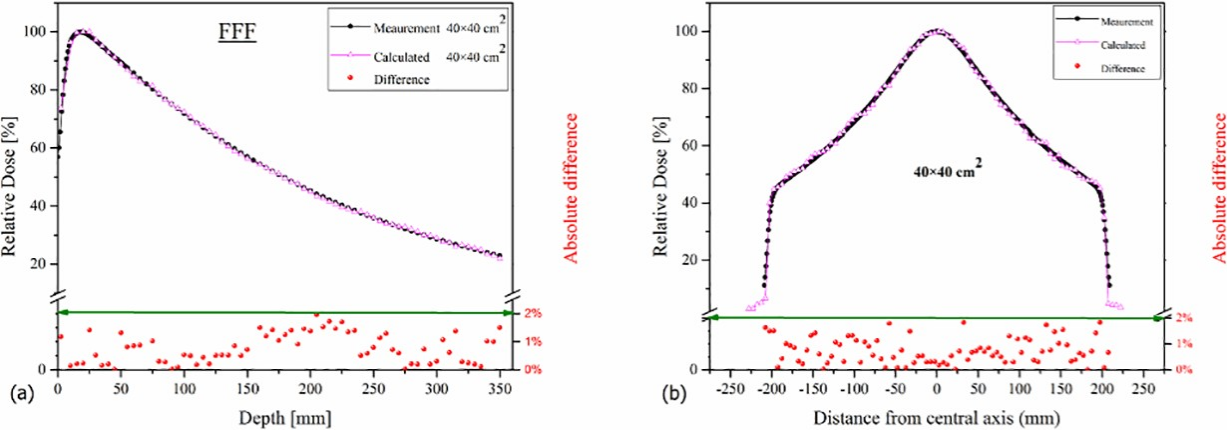
Validation of Monte Carlo simulation for 40×40 cm^2^ field size of FFF beams. a) depth-dose curve and b) beam profile in voxels with dimension of 5×5×5 mm^3^ at depth of maximum dose. Points under the green line show the absolute difference between measurement and calculation. The Monte Carlo statistical uncertainty for each point is 1% to which is not shown in figures.

The results of the optimum electron parameters for both depth-dose and beam profiles are illustrated in Fig 2 which shows the Monte Carlo validation in the case of Flat6MV and FFF beam for 10×10 and 20×20 cm^2^ field sizes. Additionally, Fig 3 shows the validation of the simulation for 40×40 cm^2^ field sizes of FFF beams. Depth dose curves were normalized to maximum dose and beam profiles at depth of maximum dose (19 mm for FFF and 16 mm for Flat6MV) were normalized to their maximum value on the central axis. The absolute difference between measurements and calculations was less than 2% for depth doses and beam profiles. The mean electron energy for the FFF beam was 8.8 MeV and 7.5 MeV for Flat6MV, the spread energy and spot size of the selected Gaussian distribution source were 0.4 MeV and 1mm, respectively.

### Depth dose

**Fig 4:**
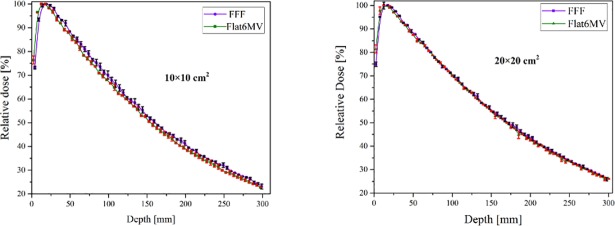
Monte Carlo calculated depth-dose of FFF and Flat6MV beams. For two field size of 10×10 and 20×20 cm^2^ in voxels with dimension of 5×5×5 mm^3^. Bars represent the uncertainty of calculation which are less than 1%.

The calculated depth dose on the central axis of both configurations of FFF and Flat6MV for two filed sizes is plotted in Fig 4. The penetration of both beam modalities is evaluated in Table 1 by the D_20_/D_10_ ratio. Overall this ratio increases by increasing the field size from 10 to 20 cm (about 7% for Flat6MV and 5% for the FFF beam). The dose rate of the FFF beam was 2.8 (2.96) times higher than for the flattened beam for a field size of 10×10 (20×20) cm^2^.

**Table 1:** Calculated D_20_/D_10_ in FFF and Flat6MV mode for two different field sizes.

**Field size (cm**^**2**^**)**	**D**_**20**_**/D**_**10**_	
	FFF	Flat6MV
**10×10**	0.587	0.578
**20×20**	0.614	0.614

D20/D10, dose ratio at a depth of 20 cm to a dose at a depth of 10 cm.

### Beam profile and FFF beam parameters

**Fig 5:**
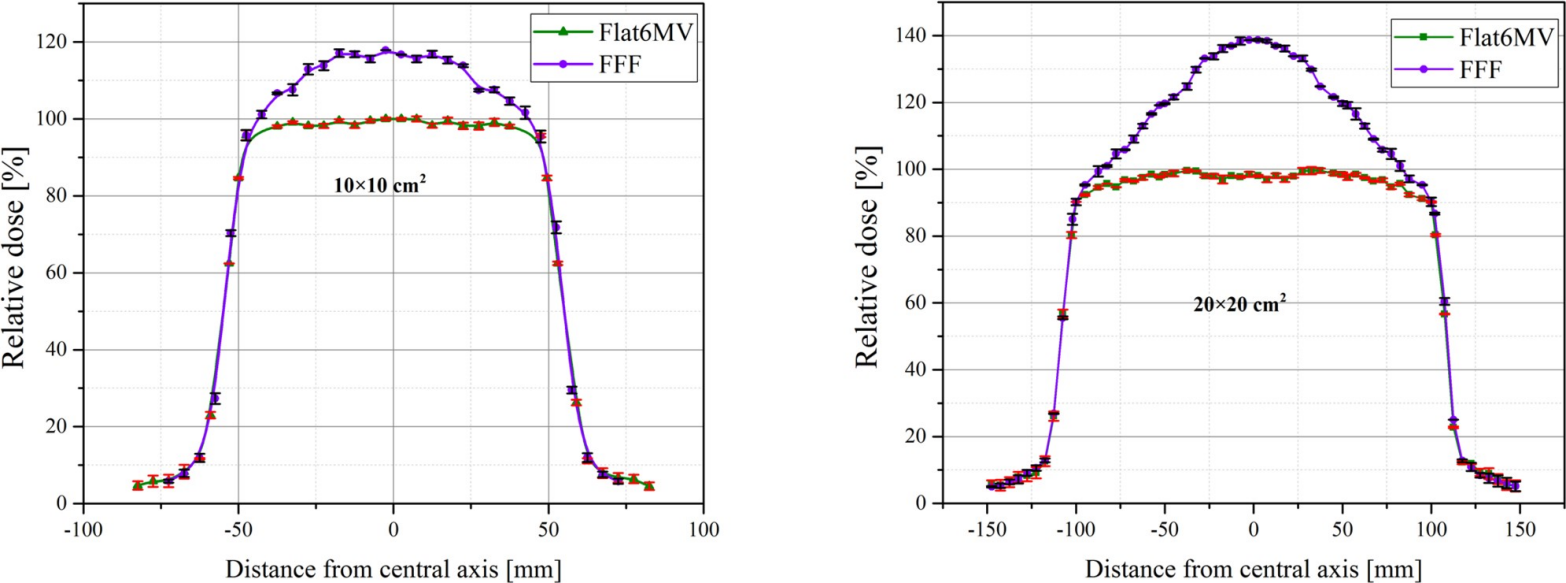
Comparison of Monte Carlo calculated beam profiles of FFF and Flat6MV. For two field sizes at depth of 10 cm in voxels with dimension of 5×5×5 mm^3^. Bars represent the uncertainty of calculation which are between 1% and 1.6%.

Fig 5 shows the comparison of the normalized beam profiles for the FFF and Flat beams for two open field sizes at depth of 10 cm. Table 2 evaluates the 20–80% penumbra for the flattened and unflattened beams. The FFF beams have a smaller penumbra because these beams have softer spectrum and also less scattering. The penumbra of the 10 cm square open field size of flattened beam was 0.4 mm larger than for the corresponding FFF beam and this difference was 0.2 mm for 20 cm square open field size.

**Table 2:** Calculated penumbra for 10 and 20 cm field side at a depth of 10 cm.

**Field size** **(cm**^**2**^**)**	**Penumbra (mm)**	
	FFF	Flat6MV
**10×10**	9.35	9.75
**20×20**	11.57	11.77

Unflatness, slope, peak position and symmetry of the FFF beam profile for three field sizes are reported in Tables 3–5. Unflatness decreases with increasing depth (about 1.5% for 10×10 cm^2^, 2.7% for 20×20 cm^2^ and 9.9% for 40×40 cm^2^ when depth increases from 1.9 to 20 cm).

**Table 3:** Calculated unflatness of the FFF beam for 10, 20 and 40 cm square fields.

Depth (cm)	Unflatness		
	10×10 cm^2^	20×20 cm^2^	40×40 cm^2^
1.9 (d_max_)	1.150	1.379	1.893
5	1.141	1.370	1.862
10	1.137	1.347	1.804
20	1.133	1.341	1.705

**Table 4:** Calculated slope of the FFF beam for 10, 20 and 40 cm square fields.

Depth (cm)	Slope		
	10×10 cm^2^	20×20 cm^2^	40×40 cm^2^
1.9 (d_max_)	3.91	4.08	3.061
5	3.37	3.58	3.037
10	2.86	2.73	3.005
20	1.08	1.71	2.901

**Table 5:** Peak position and symmetry of simulated beam profiles for 10, 20 and 40 cm square fields at depth of 10 cm.

Parameter	Field size		
	10×10 cm^2^	20×20 cm^2^	40×40 cm^2^
**Peak position**	-0.34 mm	0.25 mm	0.3 mm
**Symmetry**	2.7%	1.4%	1.1%

The slope parameter [33] for three field sizes is reported as average value between left and right slopes in Table 4. Similar to the unflatness, the slope decreases with increasing depth (from 1.9 to 20 mm depth, the decrease amounts to 72%, 58% and 5% for the 10, 20 and 40 cm square field, respectively).

Table 5 reports symmetry and peak position for the FFF beams. It should be mentioned that the possible differences may be because of statistical uncertainties of simulation and the definition of the normalization factor.

### Surface dose and buildup region

Table 6 evaluates the delivered dose in the buildup region for both configurations. It reports the calculated relative buildup dose normalized to D_max_ on the central axis and at SSD = 100 cm for each corresponding open field. It is clear that the FFF beam creates less surface dose because of less scattering compared to the flattened beam. According to Table 6 the surface dose of the FFF beam was about 21% less than for the Flat6MV for open field 10×10 cm^2^ and 20% less for 20×20 cm^2^. It is remarkable to mention that the calculated surface dose was obtained in voxels with dimension of 1×1×1 mm^3^ therefore this results are not similar to results of Fig 2 which in the voxels had 5×5×5 mm^3^ dimension.

**Table 6:** Comparison of the calculated relative surface and buildup dose of the FFF with the Flat6MV mode in voxels with dimension of 1×1×1 mm^3^.

**Buildup thickness (mm)**	**Field size (cm**^**2**^**)**			
	10×10		20×20	
	FFF	Flat6MV	FFF	Flat6MV
**1 (Surface)**	42.34	53.58	47.99	60.19
**5**	86.79	83.28	82.57	89.39
**10**	96.91	98.69	95.91	99.88

### Photon beam spectra

**Fig 6:**
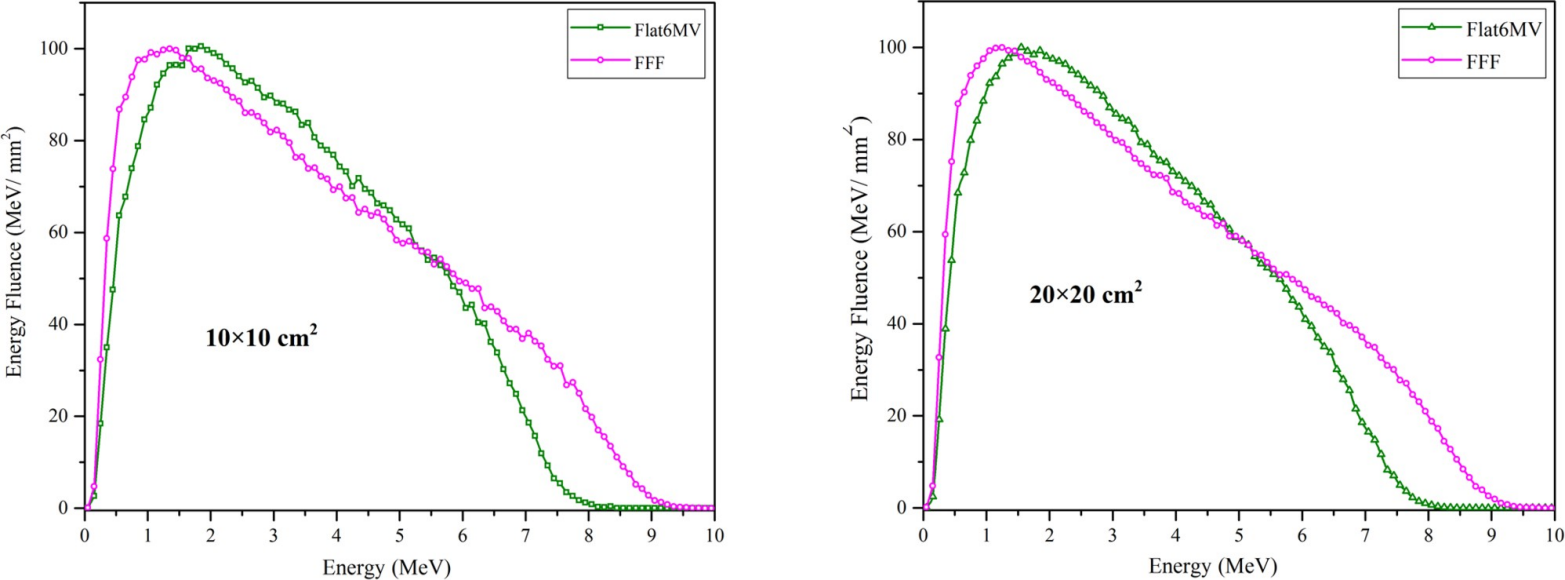
Comparison of Monte Carlo calculated photon energy fluence of FFF and Flat6MV. For two field sizes at depth of 10 cm into intervals of 0.1 MeV.

Fig 6 illustrates the photon energy distributions normalized to their maximum value (100%). Although the primary electrons in Flat6MV configuration have lower energy (7.5 MeV) compared to FFF (8.8 MeV), the FFF mode has a larger photon distribution at low energies (< = 1.55 MeV), which shows that the flattening filter acts as an energy filter for photons and the mean energy was higher for the flat 6 MV beams. The mean energy of photons of the 10×10 cm^2^ field size was 1.91/2.14 MeV for FFF / flat 6 MV beams and was 1.87 / 2.06 MeV for 20×20 cm^2^.

### Electron contamination

**Fig 7:**
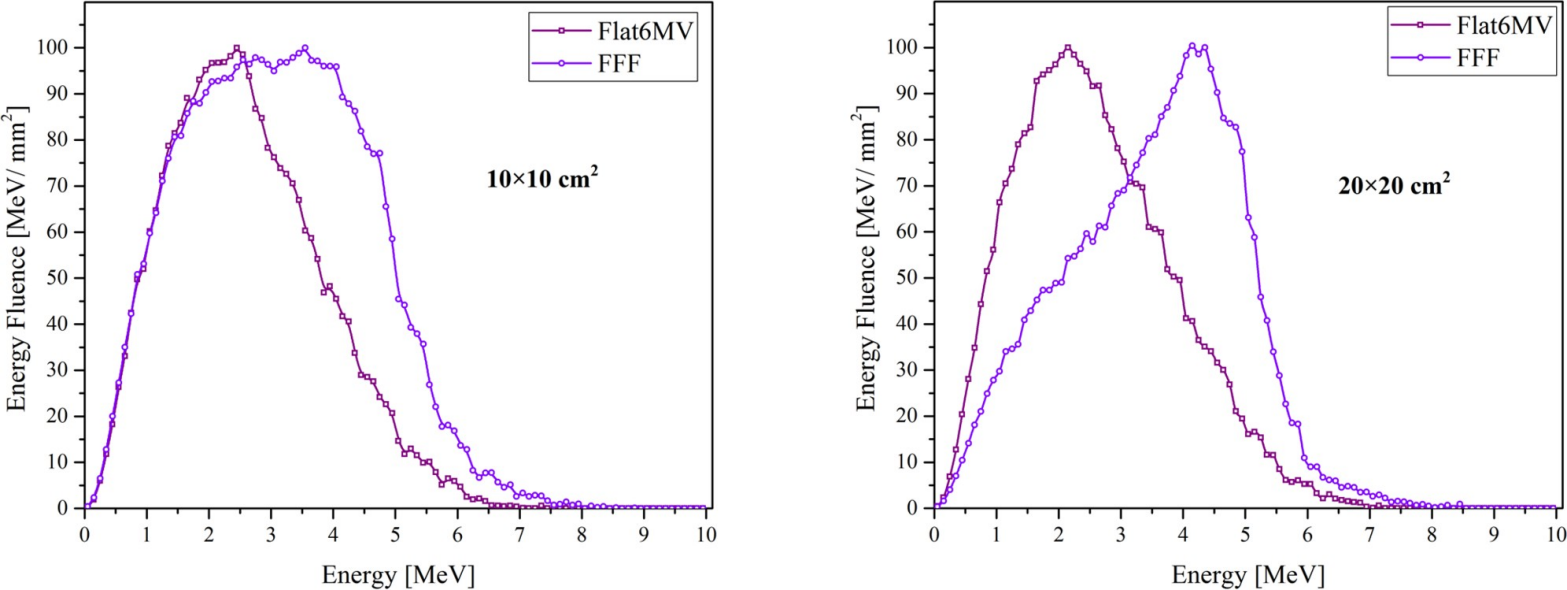
Comparison of the Monte Carlo calculated contaminant-electron energy fluence of FFF and Flat6MV. For two field sizes at the surface of the water phantom into intervals of 0.1 MeV which normalized to their maximum value (100%).

Fig 7 illustrates the electron contaminant energy fluence at the surface of the water phantom for both beam facilities. The electron mean energy of the FFF beam for the 10×10 (20×20) cm^2^ field was 3.12 (3.53) MeV and for the flat6MV 2.63 (2.61) MeV. This mean energy of the electron and their fluence can have a considerable contribution in increasing the surface dose therefore, in the Table 7 reports the contribution of contaminant-electron in surface dose which is increase with field size and shows that the flattening filter and the air are the main sources of the electron contamination.

**Table 7:** The contribution of electron contamination dose in the total surface dose.

Field size	FFF	Flat6MV
**10×10 cm**^**2**^	42.1%	52.83%
**20×20 cm**^**2**^	57.86%	60.72%

All values are normalized to maximum deposited dose of all particles on the central axis

**Fig 8:**
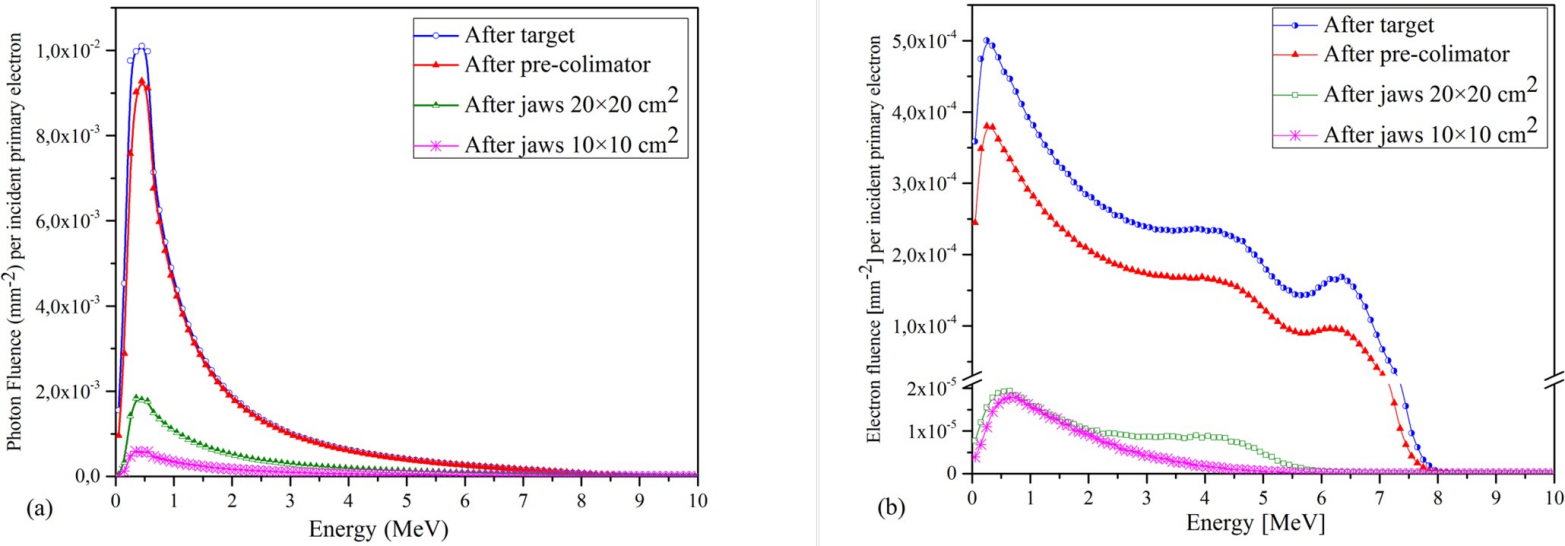
Effect of the linac head components on the photon and electron spectrum. a) photon. b) contaminant-electron fluence per incident primary electron at different stages of the beam line.

To discuss the effect of the components of the linac head on the photon spectrum, Fig 8 shows the photon and contaminant-electron fluences per incident primary electron after the target, pre-collimator, and jaws for the FFF beam. According to this figure, evidently the intensity of produced photons decreases when moving away from the target, while the mean energy increases. The mean energy of produced photons under the target, pre-collimator, and jaws for 10×10 (20×20) cm^2^ field size is 1.54 MeV and 1.61 MeV, 1.9 (1.86) MeV, respectively. On the other hand, after passing through the linac components the mean energy of the electrons decreased from 2.8 MeV under the target to 2.6 MeV, 1.8 (2.2) MeV under the pre-collimator and jaws for 10×10 (20×20) cm^2^ field, respectively.

### Off-axis change

Because of the shape of the flattening filter, the dependency of the photon beam characteristics on off-axis distance is expected to be more considerable for flattened beams than FFF beams. These dependencies on OAD for depth-dose curves of FFF and Flatt6MV beam are quantified in Table 8 by the ratio D_20_/D_10_.

**Table 8:** Comparison of calculated D_20_/D_10_ of FFF at various OAD with Flat6MV beams (20×20 cm^2^ field).

**OAD (cm)**	**D**_**20**_**/D**_**10**_	
	FFF	Flat6MV
**CAX**	0.614	0.614
**2.5**	0.6257	0.642
**5**	0.6324	0.634
**7.5**	0.6420	0.623

**Fig 9:**
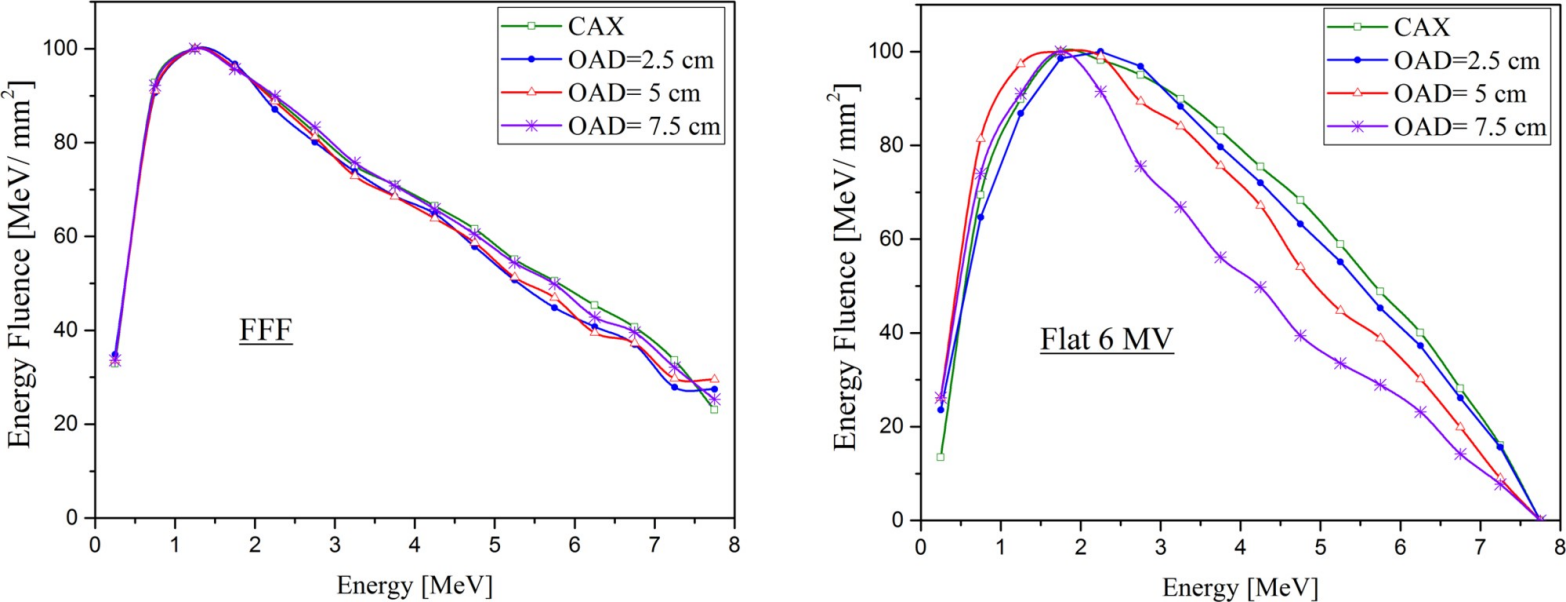
Calculated photon energy fluence on the CAX and as a function of OAX. For FFF and Flat 6 MV with 20×20 cm^2^ field size.

Table 9 reports the calculated mean energy of the FFF beam on the CAX and at different OAD for the 20×20 cm^2^ field. For FFF beams, the mean energy was 1.87, 1.85, 1.86, 1.87 MeV at OAD 0, 2.5, 5, 7.5 cm, respectively. Therefore, there is less than 0.5% variation in mean energy when the OAD increases from zero up to 7.5 cm. Fig 9 also shows that for FFF beams, the OAD has no strong effect on the mean energy of the photons, as no marked hardening effect occurs at various distance of off-axis. Nevertheless, the photon fluence was decreased by increasing the OAD, as is expected from the bremsstrahlung intensity distribution. It is clear in Fig 9 that the effect of OAD on the mean energy for the flattened beam is more considerable. Our simulations show that the mean photon energy decreases by about 12% from 2.06 MeV on the CAX to 1.81 MeV at an OAD 7.5 cm.

**Table 9:** Calculated photon mean energy of FFF beam on CAX and at difference OAX for 20×20 cm^2^ field size.

**OAD (cm)**	**Mean energy (MeV)**	
	FFF	Flat6MV
CAX	1.87	2.06
2.5	1.85	2.02
5	1.86	1.91
7.5	1.87	1.81

## Discussion

In recent years, a number of studies have studied flatting filter free modes of standard linacs with the aim to dosimetrically characterize the FFF beams and find the advantages and disadvantages of this model. Most studies have focused on the Varian and Elekta linacs, for which the technical implementation of the FFF mode is somewhat different from the Siemens approach. The present study has therefore focused on the Siemens Artiste FFF 7 MV and Flat 6 MV beam lines, both of which are in routine use at our institution. The Monte Carlo model created in Geant4 could reproduce the measured dosimetric data and yield further insight into spectral properties, surface dose and off-axis effects which are difficult to obtain experimentally.

By adjusting the histories in each calculation, the statistical uncertainty for all point in PDDs and in dosimetric field size of beams profiles were less than 1%. The statistical uncertainty for penumbra and out of dosimitric field size region was less than 1.6%.

One of the results of removing the flattering filter is increasing the dose rate. Most previous works reported an increase in dose rate by factor of 2 [36–37] who worked on Siemens ONCOR Avant-Garde and Siemens Primus linacs, respectively. For the other vendors, recently Sangeetha et al. [38] reported a relative dose rate of 2.52 (2.60) for the 10×10 cm^2^ (20×20 cm^2^) field for a Varian 600C/D and Dalaryd et al [39], observed that the dose rate of the FFF beams increased by a factor of 2.23 for an ElektaPrecise. Notably, the values for our Siemens Artiste were larger than previous ones with dose rate of 2.8 (2.96) for the 10×10 cm^2^ (20×20 cm^2^) fields.

Yarahmadi et al. [40] evaluated the penumbral width of the VarianClinac2100 linear accelerator using the EGSnrc/ BEAMnrc Monte Carlo code and reported a 0.3 mm /0.2 mm reduction in penumbra for 10×10 cm^2^/20×20 cm^2^ fields by removing the flattening filter. Comparing this with our work this reduction was 0.1 mm less than our results for 10×10 cm^2^ field size while for the 20×20 cm^2^ field size had a similar value.

It is clear that the FFF beam creates less surface dose because of less scattering, less electron contamination (Table 7) and higher primary electron energy compared to the flattened beam. Our obtained relative surface dose of the flat 6 MV beam line tended to decrease by about 21% (22%) for 10×10 cm^2^ (20×20 cm^2^) field size when the flattening filter was removed from the beam line. Contrarily, the Mohammed et al. [41] showed an increase in relative surface dose by about 19% (12%) for 10×10 cm^2^ (20×20 cm^2^) field size by removing the flattening filter from a Varian 2100 linac. For the Siemens FFF, our results converge with the results of Sigamani and Nambiray [3] who evaluated the buildup region and surface dose the Siemens Artiste 7MV-FFF and 6MV flattened photon beams using GafChromic film and two different dosimeters. About the contribution of contaminant-electrons in surface dose, our results reported that surface dose increased with increasing the field size and decreased with removing the flattening filter, which is in agreement with other studies [42, 43] with a little difference that can be due to the difference of the linacs.

Comparing our data with previous work, we find an elevated photon mean energy at the isocenter (Table 10), which distinguishes the FFF 7 MV and flat 6 MV beam line from the other linac models. In all works which are mentioned in this table, the mean energy of photon beam decreased by removing the flattening filter, similar to present work. However, for the Siemens linac this decrease is less pronounced (FFF mean energy 89% of flat beam mean energy) than for all other vendors (ratio FFF/flat mean energy 69%– 85% for 10 cm square fields). Therefore, this difference of Artiste II in mean energy was clearly observed in the depth-dose curve in which the maximum dose was obtained at a deeper position and the value of penetration (D_20_/D_10_) was higher than in previous studies.

**Table 10:** Photon mean energy at isocenter for square field sizes of 10 and 20 cm, overview of previous studies.

**Author**	**Vendor**	**FFF 7 MV**		**Flat 6 MV**	
		10×10 cm^2^	20×20 cm^2^	10×10 cm^2^	20×20 cm^2^
Mesbahi et al., 2007 ^[^^44^^]^	Elekta SL-25 linac	1.47	1.44	1.73	1.71
Vassiliev et al., 2006 ^[^^45^^]^	Varian Clinac 2100	1.22	1.22	1.77	1.77
Mesbahi, 2007 ^[^^46^^]^	Varian Clinac 21EX	1.32	1.31	1.76	1.64
Yarahmadi et al., 2013 ^[^^40^^]^	VarianClinac2100	1.26	1.26	1.81	1.81
Kajaria et al., 2017 ^[^^47^^]^	Varian Clinic 600 unique	-	1.23	-	1.52
Czarneckia et al.,2017 ^[^^20^^]^	Siemens KD	1.839	-	-	-
Present work	Siemens Artiste	1.9	1.87	2.14	2.06

Regarding the beam profiles and normalization, we have shown that it is possible to apply the renormalization method [33] to the Siemens FFF 7 MV and Flat 6 MV beams. This is not *a priori* trivial, because this method was developed for Varian FFF beams which do not differ from the flat beam except in the omission of the flattening filter. Therefore, the FFF beams are then renormalized to their “identical” flat counterpart. In our case, the energy of the FFF beam is adjusted so that the two beam lines are no longer intrinsically identical, which might result in different normalization, penumbra, and other characteristics. On the other hand, the FFF beam is matched to the flat beam regarding the depth-dose curve. It might therefore be useful to consider the two beam lines as “pdd-matched”. In fact, this would result in more similar spectral and absorption properties than for an implementation of FFF energies which just lacks a flattening filter. As our results show, the “shoulder point” or “renormalization method” can successfully be applied to this implementation of a pdd-matched FFF beam line as well, so that a good match both of the pdd and the profile properties of the two beam lines can be achieved.

## Conclusions

The Geant4toolkit was selected as a simulation code for the simulation the Siemens Artiste FFF 7 MV and flat 6 MV beam lines. All simulation results had good agreement with experimental results and allowed finding the best primary electron parameters. The removal of the flattening filter resulted in a higher dose rate, smaller penumbra, less surface dose and considerably less dependency on off-axis distance, in particular regarding the photon spectra. The normalization method could be applied to normalize the pdd-matched FFF beam to the corresponding flat beam.
